# Metabolism/Immunity Dual‐Regulation Thermogels Potentiating Immunotherapy of Glioblastoma Through Lactate‐Excretion Inhibition and PD‐1/PD‐L1 Blockade

**DOI:** 10.1002/advs.202310163

**Published:** 2024-03-09

**Authors:** Tianliang Li, Dan Xu, Zhao Ruan, Jie Zhou, Wenbo Sun, Bo Rao, Haibo Xu

**Affiliations:** ^1^ Department of Radiology Zhongnan Hospital of Wuhan University 169 Donghu Road Wuhan 430071 China; ^2^ Department of Nuclear Medicine Zhongnan Hospital of Wuhan University 169 Donghu Road Wuhan 430071 China

**Keywords:** drug‐loading thermogels, glioblastoma, immune checkpoint, lactate, vaccines

## Abstract

Intrinsic immunosuppressive tumor microenvironment (ITM) and insufficient tumor infiltration of T cells severely impede the progress of glioblastoma (GBM) immunotherapy. In this study, it is identify that inhibiting the expression of glucose transporter 1 (GLUT1) can facilitate the prevention of lactate excretion from tumor glycolysis, which significantly alleviates the lactate‐driven ITM by reducing immunosuppressive tumor‐associated macrophages (TAMs) and regulatory T cells (Tregs). Simultaneously, the findings show that the generated inflammatory cytokine IFN‐γ during immune activation aggravates the immune escape by upregulating immune checkpoint programmed death‐ligand 1 (PD‐L1) in tumor cells and TAMs. Therefore, an injectable thermogel loaded with a GLUT1 inhibitor BAY‐876 and a PD‐1/PD‐L1 blocker BMS‐1 (Gel@B‐B) for dual‐regulation of metabolism and immunity of GBM is developed. Consequently, in situ injection of Gel@B‐B significantly delays tumor growth and prolongs the survival of the orthotopic GBM mouse model. By actively exposing tumor antigens to antigen‐presenting cells, the GBM vaccine combined with Gel@B‐B is found to significantly increase the fraction of effector T cells (Th1/CTLs) in the tumor microenvironment, thereby remarkably mitigating tumor recurrence long‐term. This study may provide a promising strategy for GBM immunotherapy.

## Graphical abstract

Intrinsic immunosuppressive tumor microenvironment and insufficient tumor infiltration of T cells severely impede the progress of glioblastoma (GBM) immunotherapy. Herein, an injectable thermogel loaded with a GLUT1 inhibitor and a PD‐1/PD‐L1 blocker is fabricated for dual‐regulating the metabolism/immunity of GBM mice. Further combined with GBM vaccines significantly increased tumor infiltration of effector T cells (Th1/CTLs), delayed tumor growth, and prolonged survival.

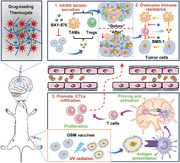

## 1 Introduction

Glioblastoma (GBM) is one of the most aggressive and destructive malignant brain tumors, with a low median survival period of 12–15 months.^[^ ^1^ ^]^ A distinctive feature of GBM is recurrence after surgical resection, radiotherapy, and chemotherapy.^[^ ^2^, ^3^, ^4^ ^]^ Immunotherapy, including immune checkpoint blockade, vaccine, and chimeric antigen receptor T‐cell therapy, has attracted great attention for systematical antitumor immunity.^[^ ^5^, ^6^, ^7^ ^]^ However, there are almost no approved and successful treatment options for GBM, which is mainly due to the intrinsic immunosuppressive tumor microenvironment (ITM) and insufficient cytotoxic T lymphocytes (CTLs) infiltration.^[^ ^8^, ^9^, ^10^ ^]^ Therefore, GBM immunotherapy focuses on efficiently reversing ITM and recruiting CTLs to tumor microenvironment (TME).

Unlike normal cells, tumor cells tend to take in excessive glucose and preferentially produce metabolic by‐product lactate (LA) even in the presence of sufficient oxygen, which is known as glycolysis.^[^ ^11^ ^]^ Enrichment of LA in TME has been verified to facilitate the establishment of ITM for promoting tumor growth and immune escape.^[^ ^12^, ^13^ ^]^ One immunosuppressive feature of LA in TME is polarizing macrophages into anti‐inflammatory phenotype M2Ф, namely tumor‐associated macrophages (TAMs).^[^ ^14^, ^15^ ^]^ Previous studies identified that LA could induce the expression of M2‐related arginase 1 in TAMs and also upregulate the vascular endothelial growth factor by stabilizing hypoxia‐inducible factor 1a for tumor progression.^[^ ^16^ ^]^ Another tumor‐promoting function of LA is acting as an alternative energy source to maintain the immunosuppressive identity of regular T cells in TME.^[^ ^17^, ^18^ ^]^ Tumor cells competitively consume glucose and excrete LA into TME, inducing energy deficiency. To maintain normal proliferation and suppressor function, Tregs would metabolize LA, rather than glucose, via monocarboxylate transporter 1 (MCT1).^[^ ^19^ ^]^ By deletion of MCT1 of Tregs, tumor growth slows, and antitumor response increases. Therefore, targeting LA metabolism is believed to effectively relieve the so‐called “cold” TME of GBM.

Besides reversing ITM, another challenge of GBM immunotherapy is activating tumor‐specific immunity and recruiting CTLs to TME. Generally, a complete immune cycle is required to activate T‐cell‐based antitumor immunity, including 1) tumor cells release and expose antigen to antigen‐presenting cells (APCs); 2) APCs present tumor antigen; 3) APCs prime and activate T cells in lymph nodes; 4) CTLs infiltrate to TME; 5) CTLs recognize and kill tumor cells.^[^ ^20^, ^21^, ^22^ ^]^ Different from other kinds of tumors, GBM is characterized by the blood‐brain barrier (BBB) and lack of lymphatic drainage, which creates difficulty in cross‐presenting antigens in the immune circle.^[^ ^23^ ^]^ Supported by the recent immunological insight that the central nervous system (CNS) is immune privileged according to preclinical and clinical studies, many attempts, including chemotherapy, radiotherapy, and photodynamic/photothermal therapy, have been developed for in situ induction of tumor immunogenic cell death (ICD).^[^ ^24^, ^25^, ^26^, ^27^, ^28^, ^29^, ^30^, ^31^, ^32^, ^33^, ^34^, ^35^ ^]^ However, these measures can cause irreversible damage to the normal brain tissue. Vaccines, including inactivated tumor cells and antigen peptides, are relatively safe agents that elicit antitumor immune responses.^[^ ^36^, ^37^ ^]^ Tumor cells tend to occur ICD when exposed to UV irradiation, such as calreticulin flipped to the outside of the cell membrane for attracting the uptake of the APCs. UV‐inactivated GBM tumor cells thereby could act as vaccines to initiate antitumor immune cycle by exposing antigens, enhancing the effector T cells such as CTLs and T helper 1 (Th1) cells infiltrating into TME.

**Figure 1:**
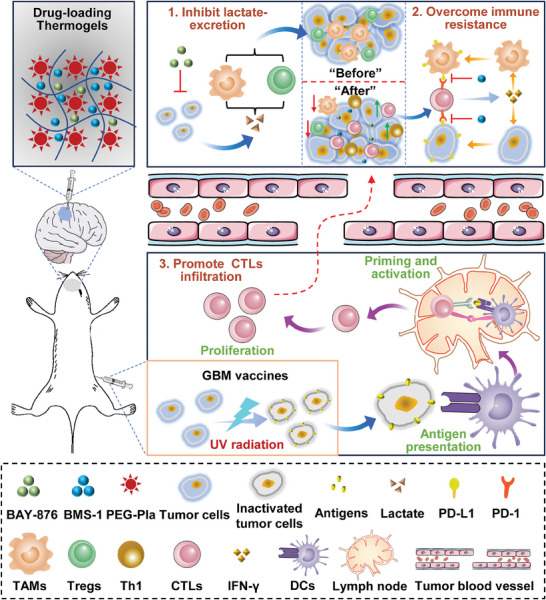
Schematic illustration of drug‐loading thermogels in alleviating ITM by inhibiting lactate excretion and overcoming immune resistance, in combination with GBM vaccines for promoting CTLs infiltrating TME.

Herein, we proposed two strategies to overcome LA‐driven ITM: blockade of lactate excretion by BAY‐876 upstream and neutralization of LA using sodium bicarbonate (SB) downstream. Compared to SB, BAY‐876 presented a more effective performance in alleviating ITM by inhibiting glucose transporter 1 (GLUT1) to prevent lactate excretion from GBM cells. Thereby, we developed an injectable thermogel (Gel@BAY) to load and controlled‐release BAY‐876. Consequently, in situ injection of Gel@BAY resulted in reduced LA content in TME, accompanied by decreased tumor infiltration of TAMs and Tregs. Further immunoassay revealed that Gel@BAY simultaneously aggravates the immune resistance by upregulating the immune checkpoint PD‐L1 in both tumor cells and TAMs, which is mainly attributed to the increased secretion of inflammatory cytokine IFN‐γ. Thereby, a PD‐1/PD‐L1 blocker BMS‐1 coupled with BAY‐876 was co‐loaded by thermogel (Gel@B‐B) to regulate the metabolism/immunity of GBM (**Figure** **1**): 1) inhibit LA‐excretion from tumor cells, and 2) overcome immune resistance of tumor cells and TAMs. Consequently, in situ injection of Gel@B‐B significantly delayed tumor growth and prolonged the survival of the orthotopic GBM mouse model. In order to further 3) enhance the effector T cells (Th1 and CTLs) infiltrating to TME, we employed the inactivated tumor cells that act as GBM vaccines to actively expose antigens to APCs, which elicited strong antitumor immune response. Combined with GBM vaccines, Gel@B‐B significantly mitigated tumor recurrence long term. Finally, we explored a rational and promising strategy for GBM immunotherapy.

## 2 Result

### 2.1 Inhibiting Lactate‐Excretion of Tumor Cells Alleviates ITM of GBM

**Figure 2:**
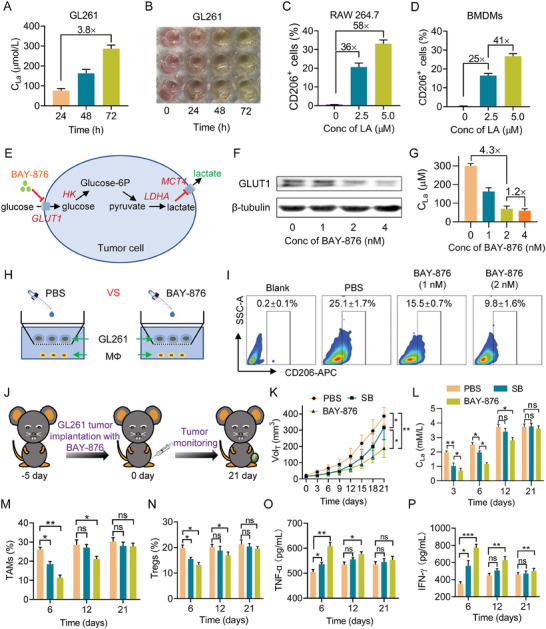
Inhibiting lactate excretion of tumor cells to alleviate ITM of GBM. A) LA concentration in medium of GL261 at different culture time (*n* = 3). B) Image of GL261‐cultured plate. C,D) FCM examination of M2Ф (CD206^+^) in LA‐treated RAW264.7 cells and BMDMs (*n* = 3). E) metabolism pathway of glucose during tumor cells glycolysis. F) Western blot assays of GLUT1 expression in. G) LA concentration in medium of BAY‐876‐treated GL261 (*n* = 3). H) In vitro M2‐polarization effect of GL261 to RAW 264.7, GL261 was treated with or without BAY‐876. I) FCM examination of M2Ф (CD206^+^) in RAW 264.7. J) Treatment of BAY‐876‐mediated GL261 tumor regression. K) Tumor growth curves of mice treated with PBS, SB, and BAY‐876 (*n* = 6). L) LA concentration in tumor tissue of mice (*n* = 3). M,N) FCM analysis of M2Ф TAMs (CD206^+^ gated in CD45^+^CD11b^+^) and Tregs (CD4^+^Foxp3^+^ gated by CD45^+^CD3^+^), *n* = 3. O,P) ELISA analysis of the intratumoral secretion of TNF‐α and IFN‐γ (*n* = 3). Data are means ± SD, ^*^ *p* < 0.05, ^**^ *p* < 0.01, ^***^ *p* < 0.001.

As LA is the metabolic by‐product of tumor glycolysis, LA assay kits were used to detect the LA content of the medium during GBM cells (GL261) culture. The level of LA gradually increased with the prolongation of culture time, and the concentration of LA in 72 h was 3.8‐fold higher than in 24 h (**Figure** **2**A). The color of the medium gradually shifted from light red (alkalescency) to distinct yellow (acidity) during different culture times, suggesting the production of LA during glycolysis of GBM cells (Figure 2B).

Next, we assessed the immunosuppressive function of LA by flow cytometry (FCM). Upon 24 hours of incubation with LA, macrophages (RAW264.7, M0Φ) can be strongly polarized into anti‐inflammatory phenotype (M2Φ). Compared to the control group, treatment with 2.5 and 5.0 µm of LA significantly augmented the M2Φ fraction by 36 and 58‐fold, respectively (Figure 2C; Figure S1, Supporting Information), confirming the immunosuppressive effect of LA. Meanwhile, LA had a comparable effect on bone marrow‐derived macrophages (BMDMs) (Figure 2D; Figure S1, Supporting Information).

LA is not only a simple metabolite of tumor glycolysis but also an important bioactive molecule.^[^ ^38^ ^]^ Thereby, we explored whether blockading the pathway of glucose metabolization could overcome its immunosuppressive effect. As shown in Figure 2E, glucose uptake through GLUT1 was the first step of the glycolysis pathway.^[^ ^39^, ^40^ ^]^ We postulated that inhibiting the expression of GLUT1 can impede tumor cells from taking in glucose and producing LA. Thereby, a GLUT1 inhibiter BAY‐876 was selected to investigate the possibility of lactate‐excretion inhibition. First, we employed a western blotting test to quantify the expression of GLUT1 in tumor cells treated with or without BAY‐876. As a result, treatment with 2 nm of BAY‐876 significantly reduced the GLUT1 expression in GL261 cells while maintaining 86% cell viability (Figure 2F; Figure S2, Supporting Information). Although 4 nm of BAY‐876 remarkably suppressed GLUT1 expression, it caused a substantial decrease in cell viability with only 35% of cells living, indicating that an appropriate concentration of BAY‐876 should be selected to inhibit lactate excretion rather than induce tumor cell death. Moreover, 2 nm of BAY‐876 showed negligible damage to the normal neuron cells, which indicated that a limited concentration of BAY‐876 was safe for the brain tissue (Figure S2, Supporting Information). Along with the GLUT1 inhibition, LA excretion from tumor cells correspondingly decreased (Figure 2G). Moreover, BAY‐876 exhibited a similar effect on human GBM cell lines of U87 and U251 (Figure S3, Supporting Information). Next, we evaluated whether treatment of BAY‐876 attenuated the M2 polarization effect of tumor cells (GL261) on macrophages (RAW 264.7) (Figure 2H). Compared to a blank group, tumor cells significantly increased the proportion of M2Φ in RAW264.7 cells from 0.2% to 25.1% (Figure 2I). After the tumor cells were treated with 1 and 2 nM of BAY‐876, the M2Φ fraction decreased to 15.5% and 9.8%, respectively, suggesting lactate excretion from tumor cells was successfully restricted through GLUT1‐inhibition.

In order to explore the in vivo ITM‐alleviation effect through inhibition of LA‐excretion, C57BL/6J mice were subcutaneously injected with 100 µL GL261 cells (4 × 10^6^) containing 2 nm of BAY‐876 or 100 µm of SB (Figure 2J). BAY‐876 was used to inhibit the release of LA from the tumor, and SB was selected to neutralize the excreted LA by tumor cells. Both SB and BAY‐876 were found to have effective tumor inhibition ability within 21 days, according to the variation of tumor volume. Compared to PBS, SB and BAY‐876 delayed ≈18.6% and 51.5% of tumor growth, respectively (Figure 2K; Figure S4, Supporting Information). Further, LA content measurement revealed that LA content in the SB group and BAY‐876 group were at the lowest level at 3 days, but gradually reached the approaching level of the PBS group at 6, 12, and 21 days (Figure 2L). Contrastively, the LA level in the BAY‐876 group was lower compared to the SB group on days 6 and 12. Considering the immunosuppressive profile of LA, the better performance in inhibition of LA‐excretion of BAY‐876 probably resulted in better antitumor performance than that of SB.

Subsequently, immunoassay was administrated to comprehensively analyze the LA‐driven ITM. The fraction of TAMs and Tregs in SB groups significantly decreased on day 6, but showed no significant change compared to the PBS group on days 12 and 21 (Figure 2M,N; Figure S5, Supporting Information). Meanwhile, the fraction of TAMs and Tregs in BAY‐876 groups presented lower than that in PBS group on days 6 and 12, while that showed no significance on day 21. ELISA assay analysis showed that the secretion amount of inflammatory TNF‐α and IFN‐γ in SB and BAY‐876 groups significantly increased on day 6, while that in SB group showed no significant change compared to the PBS group on days 12 and 21 (Figure 2O,P). The results suggested that inhibiting LA excretion by BAY‐876 was a more effective approach for alleviating LA‐driven ITM, but both SB and BAY‐876 showed reduced efficacy in the latter half of the study.

### 2.2 BAY‐876‐Loading Thermogels in Situ Reprogram ITM of GBM

**Figure 3:**
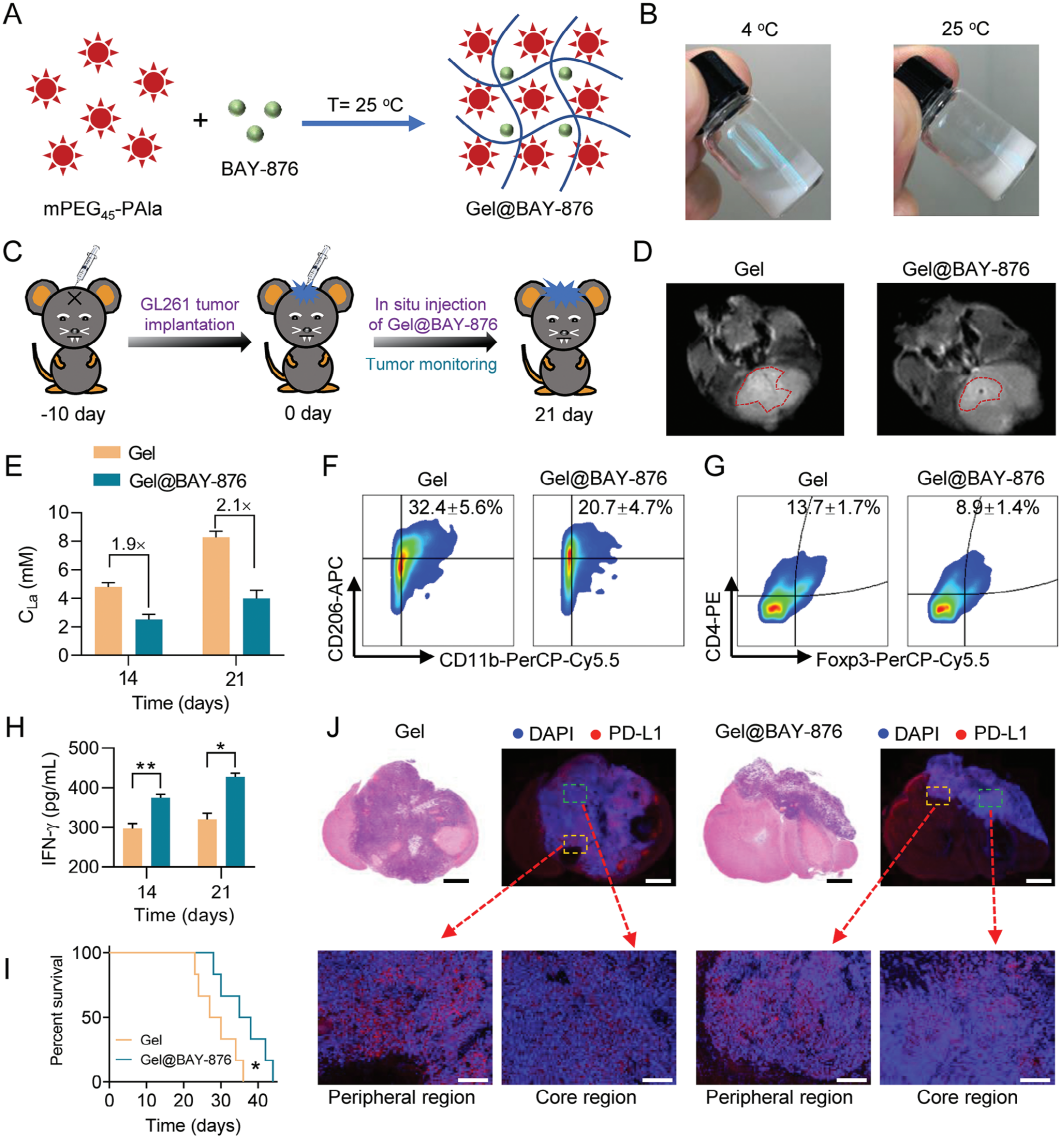
Gel@BAY in situ reprogram ITM of GBM. A) Preparation of BAY‐876‐loading polypeptide hydrogel. B) sol‐gel translation state at 4 and 25 °C. C) A treatment schedule in orthotopic GBM mice in situ injection of Gel and Gel@BAY. D) MRI images of GBM tumors at 21 days. E) LA concentration in tumor tissue of mice at 14 and 21 days (*n* = 3). F,G) FCM analysis of M2Ф TAMs (CD206^+^ gated in CD45^+^CD11b^+^) and Tregs (CD4^+^Foxp3^+^ gated by CD3^+^), *n* = 3. H) ELISA analysis of the intratumoral secretion of IFN‐γ (*n* = 3). I) Survival of mice treated with Gel and Gel@BAY within 45 days, (*p* = 0.0456, Median Survival were 28.5 and 36.5 days representively, *n* = 6). J) Immunofluorescent examination of PD‐L1 expression in tumor tissue (scale bar 1000 µm), yellow box represents peripheral region and green box represents a core region (scale bar 250 µm). Data are means ± SD, ^*^ *p* < 0.05, ^**^ *p* < 0.01.

Both SB and BAY‐b76 showed hypodynamic antitumor performance in the latter half of the study, which could be aroused due to drug leakage. Thereby, we synthesized a polypeptide PEG‐Pla as a key hydrogel molecular to efficiently load and controlled‐release BAY‐876 (**Figure** **3**A; Figure S6, Supporting Information).^[^ ^41^ ^]^ 1H‐NMR spectra confirmed the chemical structure of PEG‐Pla with the protons of a methyl group in alanine at 1.36 ppm, terminal methoxy group in mPEG, mPEG backbone at 3.40, 3.74 ppm, and methane in the backbone of Pla at 4.48 ppm (Figure S7, Supporting Information).^[^ ^42^ ^]^ FT‐IR spectra further validated the chemical structure of PEG‐Pla according to the signals at 1660 and 1543 cm^−1^ attributing to the stretching vibration of the amide bond (νC = O) of the backbone (Figure S7, Supporting Information). PEG‐Pla was dissolved in phosphate‐buffered saline (PBS, pH 7.4) with a concentration of 8.0 wt.%. The as‐prepared gel presented sol station at 4 °C and gel station at 25 °C, indicating its thermo‐responsive function of easy drug‐loading and injectable features (Figure 3B). The biodegradability of PEG‐Pla has been proved when triggered by the elastase K or α‐chymotrypsin, which confirmed that BAY‐876‐loading gel (Gel@BAY) could be used for controlled drug release in vivo investigation.^[^ ^41^ ^]^

In order to evaluate the feasibility of Gel@BAY for in situ reprograming ITM of GBM, C57BL/6J mice were injected 5 µL GL261 cells (5 × 10^5^) into the brain via microinjection syringe pump to establish an orthotopic GBM mouse model (Figure 3C). 10 days postinjection of tumor cells, Gel and Gel@BAY were in situ injected into the tumor tissues to monitor the antitumor effect and immunomodulatory ability. A magnetic resonance imaging (MRI) system was used to observe the tumor growth during the Gel and Gel@BAY treatment within 21 days. Consequently, Gel@BAY showed superior efficacy in delaying tumor growth compared to Gel (Figure 3D). Quantitative analysis revealed that the area ratio of the tumor to the brain in the Gel group was 1.8‐fold that in Gel@BAY group (Figure S8, Supporting Information). LA assay kits revealed that the level of LA in Gel@BAY group was significantly lower than that in the Gel group (Figure 3E). Specifically, at 14 and 21 days, the concentration of LA in Gel group was ≈1.72‐ and 1.59‐fold higher than that in Gel@BAY group, respectively.

Due to a significant reduction of LA content in TME of Gel@BAY‐treated mice, we assessed the tumor infiltration of immunosuppressive TAMs and Tregs by FCM, finding that at 14 days, treatment of Gel@BAY significantly reduced the fraction of TAMs and Tregs in TME (Figure 3F,G). Compared to the Gel group, the ratio of TAMs and Tregs in the Gel@BAY group were reduced by 36.1% and 35.0%, respectively (Figure S9, Supporting Information), which suggested that the sustainable release of BAY‐876 enhanced the ITM‐alleviation ability. Elia assay analysis showed that inflammatory cytokine of IFN‐γ was significantly increased in the Gel@BAY group compared to the Gel group (Figure 3H).

We monitored the survival of mice treated with Gel and Gel@BAY, finding that Gel@BAY led to prolonged survival of mice within 45 days. Nonetheless, all the mice eventually died within 45 days (Figure 3I). To elucidate the underlying reason causing the death of mice, we assessed one crucial indicator of immune resistance, i.e., the immune checkpoint of PD‐L1. The brains of Gel and Gel@BAY group were excised and made into sections to observe the distribution of PD‐L1. As shown in Figure 3J, an interesting phenomenon was observed in Gel and Gel@BAY groups, where the PD‐L1 expression of the peripheral region was remarkably higher than that of the core region. Further quantitative analysis revealed that treatment of Gel@BAY induced greater PD‐L1 expression in the core region than Gel, demonstrating that Gel@BAY elicited immune escape during immune activation (Figure S10, Supporting Information).

### 2.3 IFN‐γ Induced PD‐L1 Upregulation in Tumor Cells and TAMs

**Figure 4:**
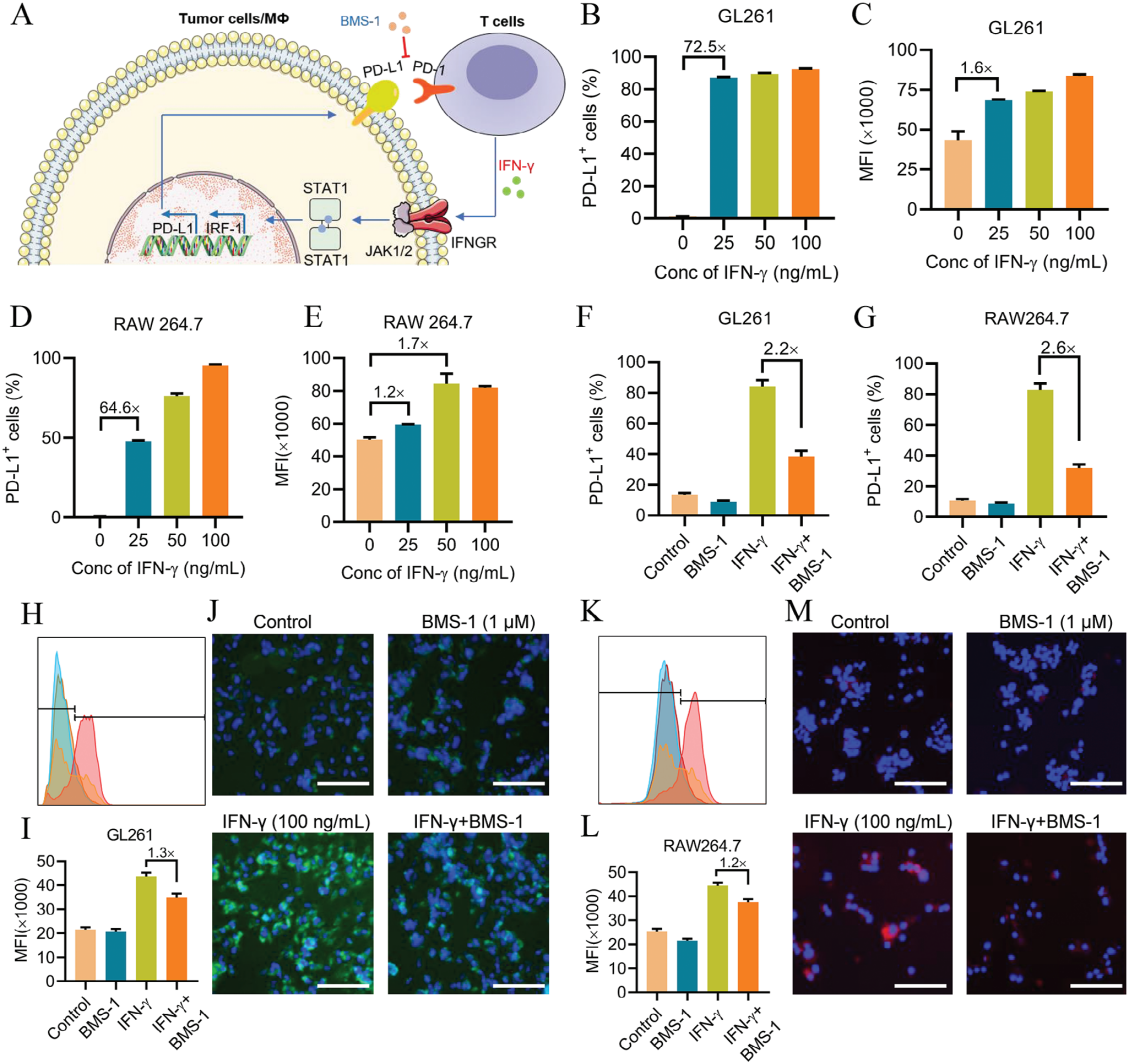
IFN‐γ‐induced PD‐L1 upregulation of tumor cells and TAMs. A) Mechanism of adaptive immune resistance between tumor cells and effector T cells in the pathway of PD‐1/PD‐L1, IFN‐γ‐inducting PD‐L1 upregulation on tumor cells and macrophages. B,C) FCM analysis and MFI of PD‐L1 expression in GL261, GL261 was incubated with different concentrations of IFN‐γ for 24 h (*n* = 3). D,E) FCM examination and MFI of PD‐L1 expression in RAW264.7, RAW264.7 was incubated with different concentrations of IFN‐γ for 24 h (*n* = 3). F–I) FCM examination and MFI of PD‐L1 expression in GL261 F) and RAW264.7 I), the cells were treated with or without IFN‐γ for 24 h (*n* = 3). H–M) CLSM observation of PD‐L1 expression in GL261 H–J) and RAW264.7 K–M), scale bar 50 µm.

Previous studies reported that the inflammatory factor of IFN‐γ could drive PD‐L1 upregulation in tumor cells through the Janus kinase (JAK)/signal transducer and activator of the transcription‐1 (STAT‐1) signaling pathway (**Figure** **4**A). We speculate that Gel@BAY aggravated inflammatory TME with an increased level of IFN‐γ in TME, thus inducing upregulation of PD‐L1. Tumor cells could exhaust effector T cells, predominantly CTLs, through the PD‐1/PD‐L1 pathway to avoid immunological recognition and clearance. In order to reveal the mechanism of adaptive immune resistance, we contrastively measured the PD‐L1 expression of tumor cells treated with or without different concentrations of IFN‐γ. FCM analysis revealed a significant upregulation of PD‐L1 expression in GL261 cells by IFN‐γ, with 25, 50, and 100 ng mL^−1^ doses increasing the proportion of PD‐L1^+^ cells from 1.2% (control group) to 87%, 89%, and 92%, respectively (Figure 4B; Figure S11, Supporting Information). Further mean fluorescence intensity (MFI) of PD‐L1^+^ cells in IFN‐γ‐treated GL261 cells gradually increased with increasing concentration of IFN‐γ (Figure 4C; Figure S11, Supporting Information).

As TAMs are important immune cells for promoting tumor growth in TME, we explored the interaction between IFN‐γ and macrophages. IFN‐γ also significantly increased the upregulation of PD‐L1 in macrophages, as demonstrated by the fraction and MFI of PD‐L1^+^ cells (Figure 4D,E; Figure S11, Supporting Information). Compared to tumor cells, macrophages showed slight insensitivity to IFN‐γ in the upregulation of PD‐L1 until the IFN‐γ concentration reached 100 ng mL^−1^, inducing over 80% PD‐L1^+^ cells. Moreover, IFN‐γ was also regarded as an important endogenous chemokine for M1‐polarization of macrophages. Therefore, IFN‐γ and LA in macrophage polarization were contrastively assessed. 100 ng mL^−1^ of IFN‐γ increased M1Ф proportion of macrophages from 4.8% (control group) to 24.5% and decreased M2Ф proportion in LA‐treated macrophages from 32.7% to 0.7% (Figure S12, Supporting Information). These results indicated that IFN‐γ had beneficial but also detrimental effects during GBM therapy.

Subsequently, we employed a PD‐1/PD‐L1 blocker (BMS‐1) to overcome the downside effect of IFN‐γ. FCM analysis revealed the PD‐L1 labeling efficacy of IFN‐γ‐treating tumor cells remarkably induced 82.8% to 31.9% by 1 µm of BMS‐1, which was attributed to the effective blocking of the combination of GL261 and α‐PD‐L1‐APC (Figure 4F). Moreover, BMS‐1 had a similar effect on macrophages (Figure 4G). Confocal laser scanning microscopy (CLSM) images showed that BMS‐1 effectively and remarkably relieved the IFN‐γ‐simulating immune escape of tumor cells and macrophages by blocking the interaction of PD‐1 and PD‐L1 (Figure 4H–M).

### 2.4 Metabolism/Immunity Dual‐Regulation Synergistically Regress GBM Growth

**Figure 5:**
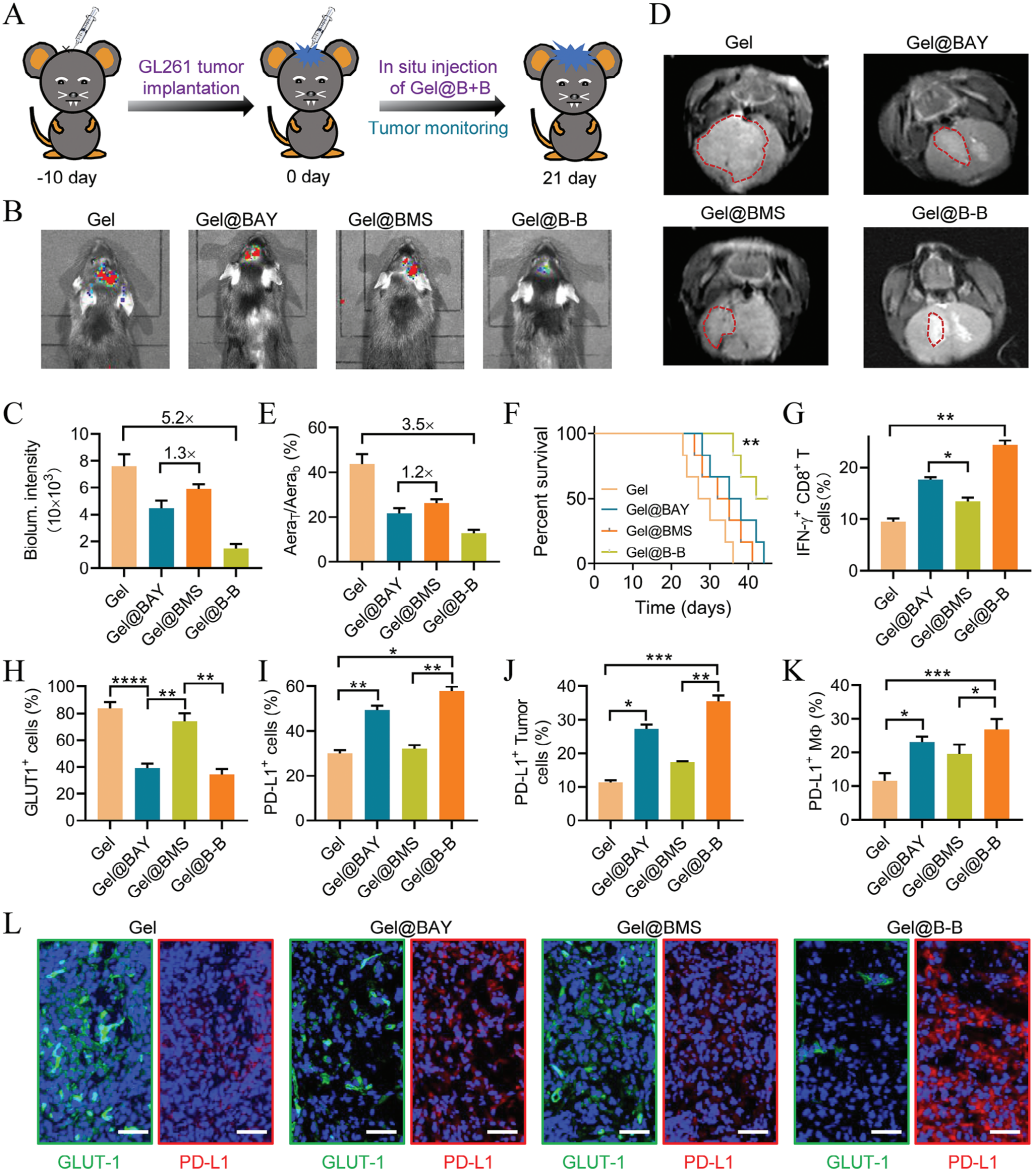
Gel@B‐B synergistically regresses GBM growth through metabolism/immunity dual‐regulation. A) treatment schedule of orthotopic GBM mice in situ injection of Gel, Gel@BAY, Gel@BMS, and Gel@B‐B. B,C) Bioluminescence images and quantified signal intensity of mice (*n* = 3). D,E) MRI images and the ratio of tumor to the brain of mice (*n* = 3). F) Survival of mice within 45 days (*p* = 0.0014, *n* = 6). G–K) FCM examination of GLUT1^+^ tumor cells (GLUT1^+^ in CD45^−^), CTLs (IFN‐γ^+^CD8^+^ in CD3^+^), PD‐L1^+^ cells (PD‐L1^+^), PD‐L1^+^ tumor cells (PD‐L1^+^ in CD45^−^), PD‐L1^+^ TAMs (PD‐L1^+^ in CD45^−^) in tumor tissues (*n* = 3). L) immunofluorescence section of tumor tissues stained with DAPI (blue), PD‐L1 (red), and GLUT1 (green), scale bar 50 µm. Data are means ± SD, ^*^ *p* < 0.05, ^**^ *p* < 0.01, ***P < 0.001, ****P < 0.0001.

The GLUT1‐inhibition by BAY‐876 exhibited the potential to alleviate the LA‐driven ITM, while PD‐1/PD‐L1 pathway blockade by BMS‐1 could reverse adaptive immune resistance. Therefore, thermogels loaded with BAY‐876 and BMS‐1 (Gel@B‐B) were developed to regress GBM growth through dual‐regulation of metabolism/ immunity. First of all, the biosafety of the drug‐loading thermogels was assessed after in situ injection into the brain tissue for 45 days. It was found that all the mice were alive within 45 days. H&E section analysis demonstrated that Gel, Gel@BAY, Gel@BMS, and Gel@B‐B just induced mechanical needle scar but no toxic effect on the brain tissues of normal mouse, suggesting the good biosafety of the drug‐loading thermogels (Figure S13, Supporting Information). C57BL/6J mice were injected 5 µL Luc^+^ GL261 cells (5 × 10^5^) into the brain via a microinjection syringe pump. The mice were in situ injected with Gel, Gel@BAY, Gel@BMS, and Gel@B‐B 10 days later (**Figure** **5**A). Tumor growth was monitored using the bioluminescence model of the Caliper IVIS Lumina II system. Consequently, Gel@B‐B exerted a synergistic effect resulting in maximum tumor suppression in all the groups. GLUT1‐inhibition of Gel@BAY showed better performance in delaying tumor growth than PD‐1/PD‐L1 blockade of Gel@BMS. Gel@BAY, Gel@BMS, and Gel@B‐B restricted ≈41.4%, 22.5%, and 80.8% of tumor growth according to the quantitative analysis of bioluminescence intensity (Figure 5B,C). Further MRI images confirmed the synergistic antitumor effect of Gel@B‐B (Figure 5D,E). Within 45 days, both Gel@BAY and Gel@BMS treatments prolonged the survival of mice; however, all mice eventually died. Due to the binary effect of reprograming ITM and overcoming immune resistance, Gel@B‐B maintained the longest survival rate, and ≈50% of mice were still alive at the end of the study (Figure 5F).

To elucidate the metabolism/immunity dual‐regulation mechanism of Gel@B‐B more comprehensively, we administrated immunoassay by FCM. Benefiting from the GLUT1‐inhibition effect of BAY‐876, the fraction of GLUT1^+^ tumor cells in Gel@BAY and Gel@B‐B group significantly decreased while that in Gel and Gel@BMS group remained high in TME (Figure 5G; Figure S14, Supporting Information). Correspondingly, intratumoral LA content was relatively lower in Gel@BAY and Gel@B‐B groups, followed by significantly decreased tumor infiltration of TAMs and Tregs compared to other groups (Figure S15, Supporting Information). Eventually, treatment of Gel@B‐B resulted in the highest fraction of CTLs (IFN‐γ^+^CD8^+^ T cells) with the most secretion amount of IFN‐γ in TME (Figure 5H; Figure S16, Supporting Information).

As the inflammatory cytokine of IFN‐γ can upregulate the expression of PD‐L1 in tumor cells and macrophages, we evaluated the PD‐L1 distribution in tumor tissue, finding that the total population of PD‐L1^+^ cells was highest in Gel@B‐B group and that in Gel@BAY and Gel@BMS group was higher than PBS group (Figure 5I; Figure S17, Supporting Information). Further analysis of PD‐L1^+^tumor cells (CD45^−^PD‐L1^+^) and PD‐L1^+^TAMs (CD45^+^CD11b^+^PD‐L1^+^) revealed that Gel@B‐B elicited the greatest PD‐L1 expression in tumor cells, and also in TAMs (Figure 5J,K; Figure S17, Supporting Information). Further immunofluorescence observation revealed that Gel@B‐B induced a remarkable reduction of GLUT1 expression for alleviating ITM, due to the incorporation of BAY‐876 (Figure 5L; Figure S18, Supporting Information). Meanwhile, we found that Gel@B‐B triggered the strongest upregulation of PD‐L1 for eliciting immune resistance, which was mainly attributed to the highest content of IFN‐γ. The results further confirmed that the incorporation of BMS‐1 to Gel@BAY enhanced the antitumor efficacy through dual‐regulation of the metabolism/immunity of GBM.

### 2.5 Gel@B‐B Combined with Tumor Vaccines Enhanced the Immunotherapy Effect of GBM

Gel@B‐B cleared the barrier of immunological recognition and elimination by reversing the “cold” GBM TME which was the enrichment of immunosuppressive cells and anti‐inflammatory cytokines. However, the BBB and the absence of lymphatic drainage posed a significant challenge for the cross‐presentation of GBM antigens between tumor cells and APCs. As this severely restricted CTLs entrance into CNS, we proposed utilizing autologous GBM vaccines to activate GBM‐specific CTLs in the lymphatic system, thus promoting CTLs infiltrating the TME.

**Figure 6:**
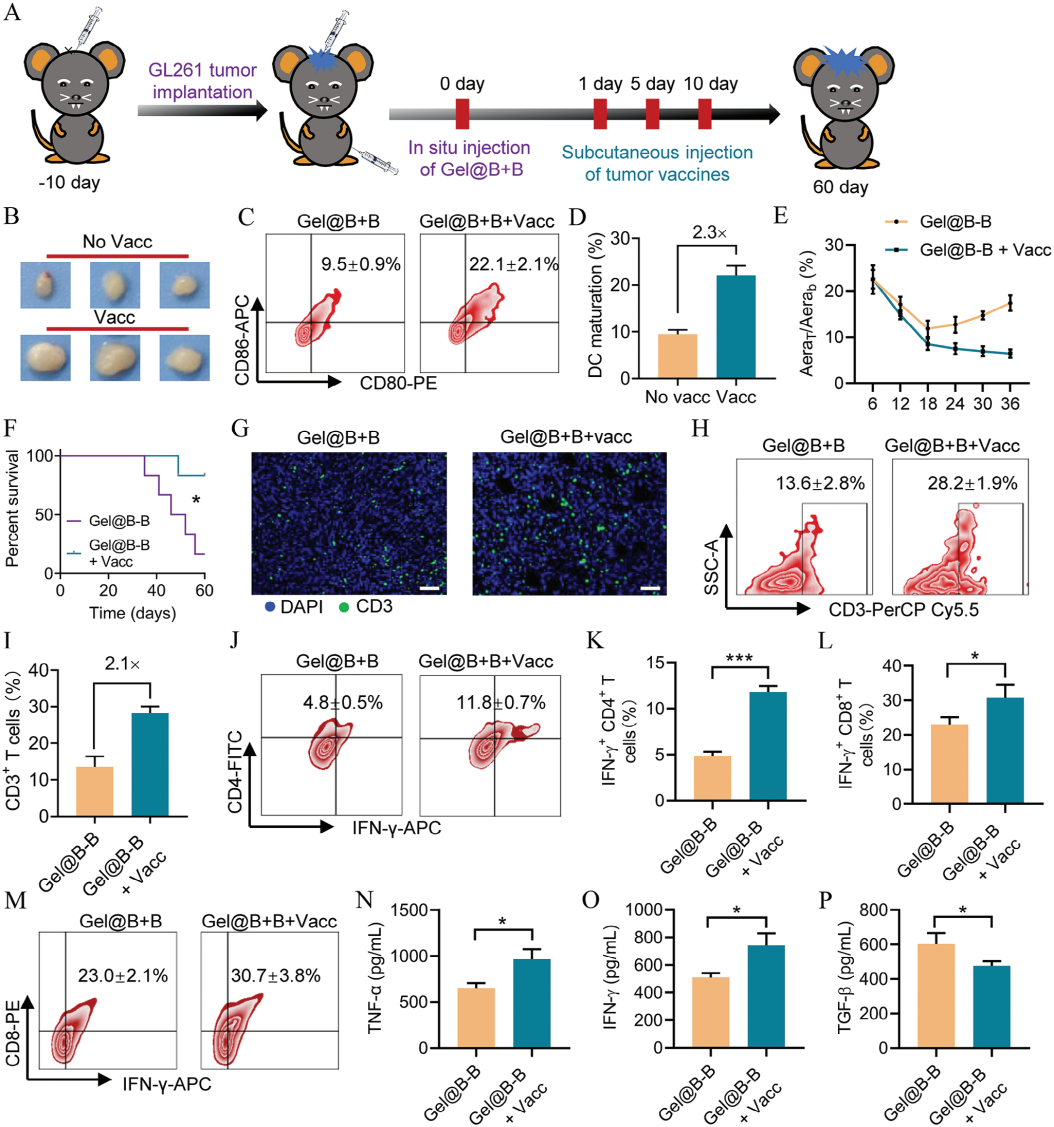
Gel@B‐B combined with tumor vaccine enhanced immunotherapy effect of GBM. A) Treatment schedule of orthotopic GBM mice in situ injection of Gel and Gel@B‐B. B) Image of LNs from mice subcutaneously injected with or without GBM vaccine. C,D) FCM examination and quantitative analysis of DC maturation (CD80^+^CD86^+^ gated in CD45^+^CD11c^+^) in LNs (*n* = 3). E) Tumor of mice imaged by MRI system. F) Survival of mice within 60 days (*p* = 0.0250, *n* = 6). G) Immunofluorescence sections of tumor tissues were stained with DAPI (blue) and CD3 (green), scale bar 50 µm. H–M) FCM examination and quantitative analysis of T cells (CD3^+^ gated in CD45^+^), Th1 (IFN‐γ^+^CD4^+^ in CD3^+^), and CTLs (IFN‐γ^+^CD8^+^ in CD3^+^), *n* = 3. N–P) ELISA analysis of the intratumoral secretion of TNF‐α, IFN‐γ, and TGF‐β (*n* = 3). Data are means ± SD, and statistical significance was calculated by t‐test. ^*^ *p* < 0.05, ^**^ *p* < 0.01, ^***^ *p* < 0.001.

GL261 cells were inactivated by exposure to UV irradiation to obtain the GBM vaccines (Figure S19, Supporting Information). The orthotopic GBM mice were injected with three doses of vaccines prior in situ injection of Gel@B‐B (**Figure** **6**A). The immune response elicited by GBM vaccines was assessed through the DC maturation efficiency in lymph nodes (LNs). After the vaccination with GBM vaccines, the size of LNs significantly expanded (Figure 6B). FCM analysis showed that the fraction of matured DCs (CD80^+^CD86^+^) in the vaccine group was ≈2.3‐fold compared to nonvaccinated group (Figure 6C,D). An MRI system was used to monitor tumor growth. Both Gel@B‐B and Gel@B‐B+vacc exhibited short‐term inhibition of tumor growth. However, the difference in tumor regression enlarged starting from 18 days, and Gel@B‐B combined with vaccines maintained a sustained antitumor effect while Gel@B‐B lost control in tumor growth (Figure 6E). The presence of GBM vaccines significantly prolonged the survival of mice in the Gel@B‐B group, with the survival rate increasing from 16.7% to 83.3% within 60 days (Figure 6F).

Immunoassay analysis was administered to elucidate the mechanisms underlying the antitumor performance of Gel@B‐B+vacc. Tumor infiltration of T cells was significantly increased in the Gel@B‐B+vacc group according to the FCM examination and immunofluorescence section staining with CD3 (Figure 6G). FCM results demonstrated that the population of T cells (CD45^+^CD3^+^) in the Gel@B‐B+vacc group was 2.1‐fold of that in the Gel@B‐B group (Figure 6H,I). Next, effector T cells of Th1 (IFN‐γ^+^CD4^+^ T cells), which were reported to promote CTLs proliferation, were evaluated. The fraction of Th1 significantly increased in the Gel@B‐B+vacc group, ≈2.4‐fold of that in the Gel@B‐B group (Figure 6J,K), thus suggesting that GBM vaccines facilitated the antitumor immunity of Gel@B‐B. Because GBM vaccines elicited systematic GBM‐specific immune responses, intratumoral CTLs were examined. FCM results showed that the fraction of CTLs in the Gel@B‐B+vacc group significantly increased compared to that in the Gel@B‐B group, thus confirming that GBM vaccination promoted CTLs infiltrating TME (Figure 6L,M). ELISA analysis revealed that treatment of Gel@B‐B+vacc significantly increased secretion of inflammatory cytokines TNF‐α and IFN‐γ and decreased anti‐inflammatory cytokines TGF‐β (Figure 6N–P).

## 3 Discussion

In our study, we identified that GBM cells could excrete metabolite by‐product LA to TME during the glycolysis, thus strongly aggravating the ITM by polarizing macrophages to tumor‐promoting M2Ф (TAMs). Except for TAMs, Tregs could utilize tumor‐excreted LA as an alternative energy source to maintain the immunosuppressive identity. In order to prove the concept, we proposed two ways to reverse the LA‐driven ITM through neutralization of LA using SB or impeding LA excretion by BAY‐876. In the short term, those two ways significantly decreased tumor infiltration of TAMs and Tregs in TME and delayed tumor growth. With time prolonging, SB and BAY‐876 gradually lost efficacy in the regulation of ITM (day 12) due to the decomposition of SB and leakage of BAY‐876. Considering BAY‐876 showed a better antitumor effect than SB, we utilized injectable thermogels (PEG‐Pla) to load and control the release of BAY‐876. Effectually, in situ, injection of Gel@BAY showed long‐term function in reprogramming ITM with significantly reduced LA content and tumor infiltration of TAMs and Tregs. Therefore, we identified a valuable strategy for GBM immunotherapy by regulating metabolism to overcome the “cold” TME of GBM.

However, we found that Gel@BAY simultaneously induced an inevitable consequence, i.e., the establishment of immune resistance during the activation of antitumor immunity. Specifically, inflammatory cytokine IFN‐γ could upregulate the expression of immune checkpoint PD‐L1 in tumor cells, thus exhausting CD8^+^ T via the pathway of PD‐1/PD‐L1. Besides tumor cells, this phenomenon was also observed in TAMs. Therefore, we found that the key cytokine IFN‐γ of antitumor immunity could exert positive effects, such as killing tumor cells and polarizing TAMs to M1Ф, and negative effect of eliciting immune escape, including upregulating PD‐L1 of tumor cells and TAMs. This phenomenon probably accounted for the eventual mortality of mice treated with Gel@BAY within 45 days. A PD‐1/PD‐L1 blocker BMS‐1 coupled with GLUT1 inhibitor BAY‐876 was co‐loaded into thermogels (Gel@B‐B) to reprogram ITM and overcome immune resistance. Consequently, Gel@B‐B showed a potentiated antitumor effect and prolonged survival of mice compared to Gel@BAY and Gel@BMS. Therefore, a synergistic strategy for the dual regulation of metabolism and immunity was established for GBM immunotherapy.

Gel@B‐B reversed ITM by inhibiting GLUT1 expression and overcoming immune resistance by blocking the PD‐1/PD‐L1 pathway, which cleared the barrier of immunological recognition and elimination. Yet, the presence of the blood‐brain barrier and the absence of lymphatic drainage led to a paucity of CTLs infiltration in GBM TME, which was another obstacle to GBM immunotherapy. Specifically, the cross‐presentation of tumor antigens was challenging for APCs. In order to promote CTLs infiltration into the TME, we proposed the utilization of GBM vaccines to actively expose tumor antigens to APCs, thus eliciting systematic GBM‐specific immune response. Through a combination of GBM vaccines, Gel@B‐B induced greater tumor infiltration of effector T cells, including Th1 and CTLs. Within 60 days, Gel@B‐B+vacc showed long‐term tumor suppression without recurrence and remarkably prolonged survival of mice compared to Gel@B‐B. Finally, It should be pointed out that the antitumor effect of Gel@B‐B+vacc system has only been verified in GBM model. The significance of our drug delivery system needs to be comprehensively recognized in other kinds of tumors.

## 4 Conclusion

This study reported the strategy for enhancing GBM immunotherapy through alleviating LA‐driven‐ITM, overcoming adaptive immune resistance, and vaccines‐mediated tumor infiltration of T cells. Inhibition of LA excretion by a GLUT1 inhibitor BAY‐876 significantly reduced immunosuppressive TAMs and Tregs infiltration to TME. Blockade the immune checkpoint pathway by a PD‐1/PD‐L1 blocker BMS‐1 effectively resisted the IFN‐γ‐induced immune escape. Through co‐loading BAY‐876 and BMS‐1 by an injectable thermogel (Gel@B‐B), synergistic antitumor effects were obtained by in situ injection of Gel@B‐B to the orthotopic GBM mouse model. Gel@B‐B cleared the barrier of immunological recognition and elimination, GBM vaccines combined with Gel@B‐B further induced more fraction of effector T cells (Th1/CTLs) infiltrate to TME. Eventually, we summarized a reasonable scheme for GBM therapy.

## 5 Experimental Section

### Materials

BAY‐876 and BMS‐1 were purchased from MedChemExpress (MCE, China). L‐Ala‐NCA (CAS: 2224‐52‐4) and mPEG_45_‐NH_2_ (CAS: 80506‐64‐5) were purchased from Aladdin (China). LA assay kits were obtained by Jianchengbio (China). CCK‐8 assays, lysed RIPA buffer, PMSF, DAPI anti‐β‐tubulin antibody, antiIgG‐FITC antibody, anti‐IgG‐Cy3 antibody, and anti‐GLUT1 antibody, anti‐PD‐L1 antibody, HRP‐conjugated anti‐rabbit IgG and antimouse IgG were purchased from Servicebio (China). Murine IFN‐γ, TNF‐α, and TGF‐β ELISA kits, a cytokine of murine IFN‐γ were provided by Neobioscience (Shenzhen, China). anti‐PD‐L1‐APC antibody, anti‐CD80‐PE antibody, anti‐MHCII‐FITC antibody, anti‐CD45‐PE antibody, anti‐CD45‐FITC antibody, anti‐CD11b‐PerCP‐Cy5.5 antibody, anti‐CD3‐PerCP‐Cy5.5 antibody, anti‐CD206‐APC antibody, anti‐CD4‐FITC antibody, anti‐Foxp3‐APC antibody, anti‐CD11b‐PerCP‐Cy5.5 antibody, anti‐IFN‐γ‐APC antibody were purchased from Liankebio (China). anti‐CD206‐APC antibody was provided by Elabscience (China).

### Cell Lines and Animals

Mouse GBM cells of GL261, Human GBM cell lines of U87 and U251, Mouse macrophage cell lines of RAW264.7 were cultured using complete medium containing Dulbecco's Modified Eagle's Medium (DMEM) supplemented with 10% (v/v) of fetal bovine serum (FBS, Gibco) and 1% penicillin/streptomycin (Gibco) at 37 °C in 5% CO_2_.

C57BL/6 mice (4–5‐week old, 18–20 g, female) were obtained from the Beijing Vital River Laboratory Animal Technology Co., Ltd. (Beijing, China). All animal procedures were carried out under the guidelines approved by the Institutional Animal Care and Use Committee (IACUC) of Wuhan University.

### In Vitro Assessment of GL261 Glycolysis

To detect the LA excretion from tumor cells, GL261, U87, and U251 cells were cultured in 96‐well plates (1 × 10^4^/well) for 24, 36, and 72 h. The medium was collected to measure the LA concentration using LA assay kits according to the manufacturer's procedure. At the end of the study, the plates were imaged by camera to observe the color change of the medium.

To evaluate the LA‐excretion inhibition ability of BAY‐876, GL261 cells were cultured in 96‐well plates (1 × 10^4^/well) with the medium containing 0, 1, 2, 4 ng mL^−1^ of BAY‐876 for 72 h. The medium was collected to test the LA concentration using LA assay kits. At the end of the study, the viability of the cells was tested using the CCK‐8 assays. The viability of normal neuron cells (SW‐10) was contrastively measured by CCK‐8 assays after treating with 0, 1, 2, 4 ng mL^−1^ of BAY‐876 for 24 h.

To verify the GLUT1‐expression inhibition ability of BAY‐876, GL261 cells were cultured in six‐well plates (5 × 10^5^/well) with the medium containing 0, 1, 2, 4 ng mL^−1^ of BAY‐876. The cells were collected and lysed RIPA buffer containing 1 mm of PMSF for western blotting analysis. The lytic cells were discarded after centrifugation at 14 000 rpm for 20 min at 4 °C, and the supernatant was mixed with 5× gel loading buffer at a volume ratio of 1:4 and boiled at 100 °C for 10 min. Equal quantities of samples were subjected to gel electrophoresis using SDS‐PAGE and transferred onto a PVDF membrane. After blocking with 5% nonfat milk in 1 × TBST for 1 h at room temperature, the membranes were incubated with primary antibodies against β‐tubulin and GLUT1 protein, overnight at 4 °C. After incubating with HRP‐conjugated antirabbit and anti‐mouse IgG secondary antibodies for 1 h at room temperature, the membranes were visualized with chemiluminescence detection (Bio‐Rad Laboratories, USA) and analyzed with Image J (NIH, USA).

### In Vitro Adaptive Immune Resistance Evaluation of Tumor Cells and Macrophages

To explore the PD‐L1 upregulation effect of IFN‐γ on tumor cells and macrophages, GL261 cells, and RAW264.7 cells were cultured in 6‐well plates (5 × 10^5^/well) with the medium containing 0, 25.50, and 100 ng mL^−1^ of IFN‐γ. After incubation for 24 h, the cells were collected and stained by anti‐PD‐L1‐APC antibody coupled with or without 1 µm of BMS‐1 for FCM examination.

To visually observe the variation of PD‐L1 expression on tumor cells and macrophages, GL261 cells, and RAW264.7 cells were cultured in four well‐chambered bottom dishes (1 × 10^5^/well) with the medium containing 0 or 100 ng mL^−1^ of IFN‐γ for 24 h. After being stained by DAPI, anti‐PD‐L1‐APC antibody coupled with or without 1 µm of BMS‐1, the cells were imaged by a confocal scanning laser microscope (CLSM).

### In Vitro Macrophage‐Polarization Assessment

To explore the M2‐polarization of exogenous LA on macrophages, RAW264.7 cells were cultured in 6‐well plates (5 × 10^5^/well) with the medium containing 0, 2.5, and 5 µm of LA for 24 h. The cells were collected and stained by anti‐CD206‐APC antibody for FCM examination.

To explore the M2‐polarization of endogenous LA on macrophages, RAW264.7 cells were cultured in 12‐well plates (2 × 10^5^/well). The wells were then added cell culture inserts with 0.4 µm translucent high‐density PET membrane (BD Falcon, BD‐353503). GL261 cells (2 × 10^5^/well) pretreated with or without 2 ng mL^−1^ BAY‐876 were added into the cell culture insert for 24 h co‐incubation with RAW264.7 cells. RAW264.7 cells were collected and stained by anti‐CD206‐APC antibody for FCM examination.

To explore the M1‐polarization of IFN‐γ, RAW264.7 cells were cultured in 6‐well plates (5 × 10^5^/well). The medium contained 0, 50, and 100 ng mL^−1^ of IFN‐γ coupled with or without 2.5 or 5 µm of LA. After incubation for 24 h, the cells were collected and stained by anti‐CD80‐PE antibody and anti‐MHCII‐FITC antibody for M1Ф analysis, and stained anti‐CD206‐APC antibody for M2Ф analysis.

### In Vivo ITM‐Alleviation Effect of Lactate‐Excretion Inhibition

C57BL/6J mice were subcutaneously injected 150 µL GL261 cells (4 × 10^6^) containing with 100 µm of sodium bicarbonate (SB) or 2 nm of BAY‐876. After 5 days post‐injection of tumor cells, the tumor growth was starting to be monitored, and the tumor volume was calculated according to the formula V = L*W*W/2 (L represented the longest dimension, and W represented the shortest dimension).

To measure the content of LA, TNF‐α, and IFN‐γ in TME, the tumor tissues were homogenized using a homogenizer. After centrifugation at 12 000 rpm for 10 min, the LA concentration was measured using LA assay kits, and the TNF‐α and IFN‐γ concentrations were measured by mouse TNF‐α and IFN‐γ ELISA kits according to the manufacturer's instructions.

To reveal the ITM‐alleviation mechanism of lactate‐excretion inhibition, the tumor tissues were dissociated into cell suspension for FCM examination. After treatment with red blood cell lysis buffer (Servicebio, G2015, China), the cells were stained with anti‐CD45‐PE antibody, anti‐CD11b‐PerCP‐Cy5.5 antibody, and anti‐CD206‐APC antibody for M2Φ analysis; anti‐CD3‐PerCP‐Cy5.5 antibody anti‐CD4‐FITC antibody, anti‐Foxp3‐APC antibody for Tregs.

### Synthesis and Characterization of PEG‐Pla

PEG‐Pla was synthesized using the previously described method. Typically, 3 mmol L‐Ala‐NCA and 0.1 mmol mPEG‐NH_2_ were added into 10 mL anhydrous N,N‐dimethylformamide (DMF) for 72 h stirring at 25 °C. After adding another 10 mL DMF, the solution was transferred into 200 mL cold diethyl ether to precipitate the polypeptide. The above‐obtained precipitate was dissolved in DMF and dialyzed against water three times (2000 mL/time, 24 h). Finally, the final polypeptide was obtained through lyophilization. PEG‐Pla was dissolved in deuterated trifluoroacetic acid for ^1^H NMR analysis and was tableted using potassium bromide (KBr) for FT‐IR analysis. Number‐average molecular weights (M*n*) and polydispersity indexes (PDIs) were detected by gel permeation chromatography (GPC).

### Preparation of Drug‐Loading Thermogels

To obtain the drug‐loading thermogels, PEG‐Pla mixed with BAY‐876 or BMS‐1 or BAY‐876+BMS‐1 was dissolved in phosphate‐buffered saline (PBS) to form Gel, Gel@BAY, Gel@BMS, and Gel@B‐B. The weight ratio of PEG‐Pla:PBS was set to 8%, and the concentration of BAY‐876, BMS‐1 was set to 10 nm and 1 µm. The sol‐gel transition behavior was imaged by the camera when the thermogels were maintained at 4 and 25^ o^C. The morphology was observed by scanning electron microscope (TESCAN CLARA).

### In Vivo Biosafety of Gel@B‐B

to assess the safety of Gel@B‐B, normal C57BL/6J mice were in situ ininjection of Gel, Gel@BAY, Gel@BMS, and Gel@B‐B into the brain tissue, respectively. Survival of mice was monitored within 45 days and the brain tissue section of all groups was analyzed by H&E staining.

### In Vivo ITM‐Reprograming of Orthotopic GBM Mouse Model

To evaluate the in vivo ITM‐reprograming effect, C57BL/6J mice were injected 5 µL GL261 or Luc^+^GL261 cells (5 × 10^5^) into the brain via microinjection syringe pump to establish an orthotopic GBM mouse model. At 10 days post‐injection of tumor cells, Gel, Gel@BAY, Gel@BMS, and Gel@B‐B were in situ injected into the tumor tissue, respectively. Tumor growth was observed by an MRI system. Survival of mice was monitored within 45 days.

To measure the content of LA, TNF‐α, and IFN‐γ in TME, the tumor tissues were homogenized using a homogenizer. The LA concentration was measured using LA assay kits, and the TNF‐α and IFN‐γ concentrations were measured by mouse TNF‐α and IFN‐γ ELISA kits according to the manufacturer's instructions.

To determine the metabolism/immune modulation of Gel@BAY, the tumor tissues were excised and made into cell suspension for immunoassay analysis. The cells were stained with anti‐CD45‐PE antibody, anti‐GLUT1 antibody + anti‐IgG‐FITC antibody for GLUT1^+^ cells analysis; anti‐CD45‐PE antibody, anti‐CD11b‐PerCP‐Cy5.5 antibody, anti‐PD‐L1‐APC antibody for PD‐L1^+^ cells analysis; anti‐CD45‐PE antibody, anti‐CD11b‐PerCP‐Cy5.5 antibody, and anti‐CD206‐APC antibody for M2Φ analysis; anti‐CD3‐PerCP‐Cy5.5 antibody, anti‐CD4‐FITC antibody, anti‐Foxp3‐APC antibody for Tregs analysis, anti‐CD3‐PerCP‐Cy5.5 antibody, anti‐CD8‐FITC antibody, anti‐IFN‐γ‐APC antibody for CTLs analysis.

To observe the GLUT1 distribution in TME, the tumor tissue section was stained with DAPI, anti‐GLUT1 antibody, and anti‐IgG‐FITC antibody for immunofluorescence observation. To observe the PD‐L1 distribution in TME, the tumor tissue section was stained with DAPI, and anti‐PD‐L1 antibody, and anti‐IgG‐Cy3 antibody for immunofluorescence observation.

### Antitumor Immunity Assessment of Gel@B‐B Combined with GBM Vaccines

To fabricate GBM vaccines, 5 × 10^6^ of GL261 cells were exposed to UV radiation for inactivation treatment. after orthotopic GBM mouse were in situ injected Gel@B‐B, the above‐prepared GBM vaccines were subcutaneously injected. Tumor volume and survival of mice were monitored within 60 days.

To evaluate the immune response elicited by Gel@B‐B+vac, the inguinal lymph nodes (LNs) and tumor tissues were excised and made into cell suspension for immunoassay analysis. The cells in LNs were stained with anti‐CD45‐FITC antibody, anti‐CD11c‐PerCP‐Cy5.5 antibody, anti‐CD86‐APC antibody, anti‐CD80‐PE antibody for DC analysis; anti‐CD45‐PE antibody, anti‐CD11b‐PerCP‐Cy5.5 antibody, and anti‐CD206‐APC antibody for M2Φ analysis; anti‐CD3‐PerCP‐Cy5.5 antibody, anti‐CD4‐FITC antibody, anti‐Foxp3‐APC antibody for Tregs analysis; anti‐CD3‐PerCP‐Cy5.5 antibody, anti‐CD4‐FITC antibody, anti‐ IFN‐γ‐APC antibody for Th1 analysis; anti‐CD3‐PerCP‐Cy5.5 antibody, anti‐CD8‐FITC antibody, anti‐IFN‐γ‐APC antibody for CTLs analysis.

To measure the intratumoral secretion of IFN‐γ, TNF‐α, and TGF‐β in TME, the tumor tissues were homogenized using a homogenizer. The IFN‐γ, TNF‐α, and TGF‐β concentrations were measured by mouse IFN‐γ, TNF‐α, and TGF‐β ELISA kits according to the manufacturer's instructions.

### Statistical Analysis

Data are means ± SD, and the statistical significance was displayed by one‐way analysis of variance (ANOVA) with Tukey's post hoc test and two‐sided unpaired Student's *t* test. Comparisons of survival rates were calculated by the log‐rank (Mantel–Cox) test. Statistical significance was set as follows: ^*^ *p* < 0.05, ^**^ *p* < 0.01, ^***^ *p* < 0.001, ^****^ *p* < 0.0001.

## Conflict of Interest

The authors declare no conflict of interest.

## Supporting information

Supporting Information

## Data Availability

The data that support the findings of this study are available from the corresponding author upon reasonable request.
