# Supplementary material for: Metabolism/Immunity Dual‐Regulation Thermogels Potentiating Immunotherapy of Glioblastoma Through Lactate‐Excretion Inhibition and PD‐1/PD‐L1 Blockade
Source: Adv Sci (Weinh). 2024 Mar 9;11(18):2310163. doi: 10.1002/advs.202310163 (PMC11095231; doi:10.1002/advs.202310163)
Supplement: Supplementary file 1 — Supporting Information [file ADVS-11-2310163-s001.pdf]

ADVANCED SCIENCE
Open Access

# Supporting Information

for *Adv. Sci.*, DOI 10.1002/advs.202310163

Metabolism/Immunity Dual-Regulation Thermogels Potentiating Immunotherapy of Glioblastoma Through Lactate-Excretion Inhibition and PD-1/PD-L1 Blockade

*Tianliang Li, Dan Xu, Zhao Ruan, Jie Zhou, Wenbo Sun, Bo Rao and Haibo Xu**

Supporting Information

**Metabolism/immunity dual-regulation thermogels potentiating immunotherapy of glioblastoma through lactate-excretion inhibition and PD-1/PD-L1 blockade**

*Tianliang Li, Dan Xu, Zhao Ruan, Jie Zhou, Wenbo Sun, Bo Rao, Haibo Xu**

T. Li, ZH. Ruan, J. Zhou, W. Sun, B. Rao, H. Xu

Department of Radiology, Zhongnan Hospital of Wuhan University, 169 Donghu Road, Wuhan 430071, China. E-mail: xuhaibo@whu.edu.cn

D. Xu

Department of Nuclear Medicine, Zhongnan Hospital of Wuhan University, 169 Donghu Road, Wuhan 430071, China.

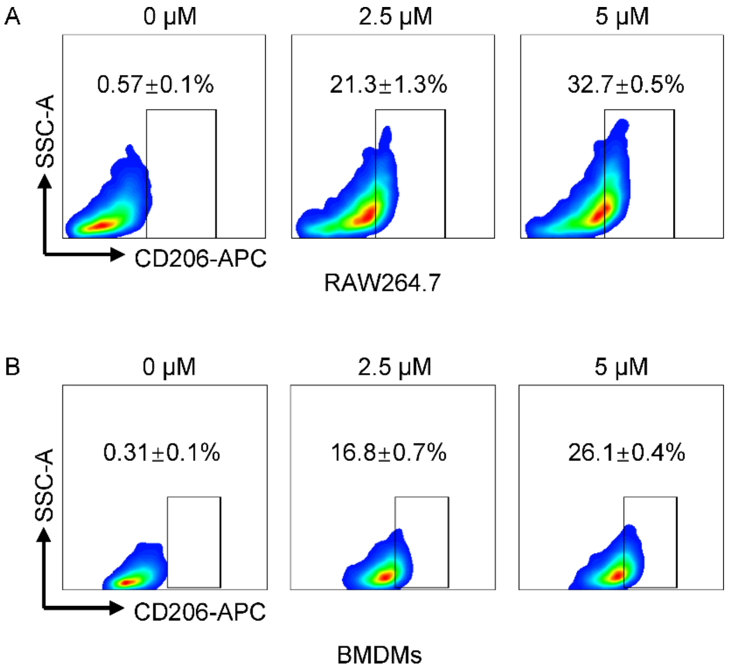


**Fig S1.** FCM examination of LA-induced M2-polarization on RAW264.7 **(A)** and BMDMs **(B)**, concentration of LA was set as 0, 2.5 and 5 μM.

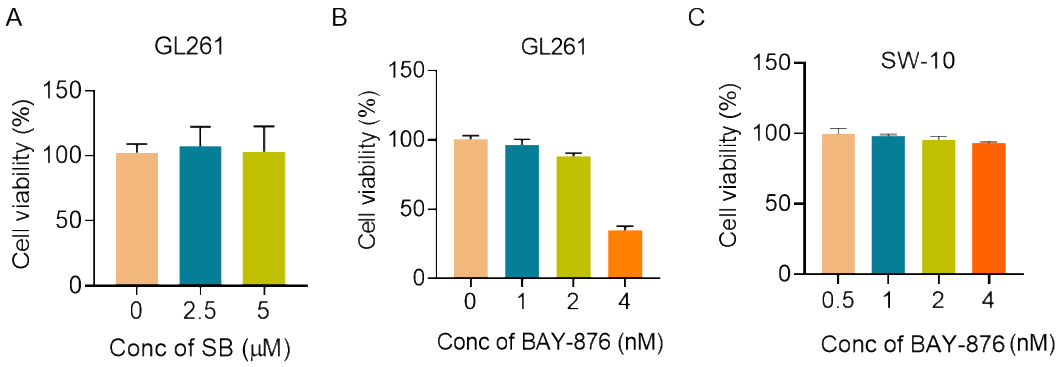


**Fig S2.** Cell viability of SB-treated **(A)**, BAY-876-treated **(B)** GL261 cells, and BAY-876-treated SW-10 cells. The cells were incubated with SB or BAY-876 for 24 hours

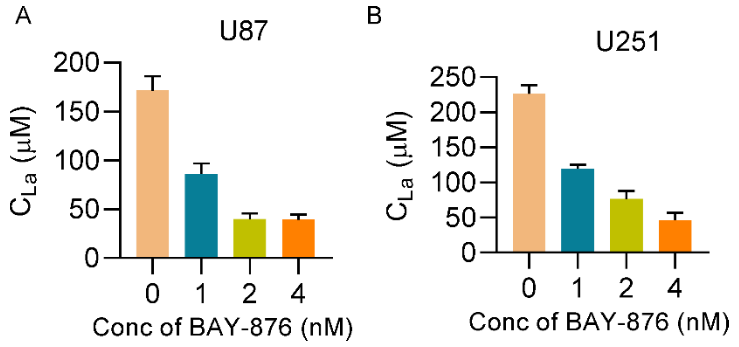


**Fig S3.** LA concentration in the medium of BAY-876-treated U87 **(A)** and U251 **(B)**, the cells were incubated with BAY-876 for 72 hours.

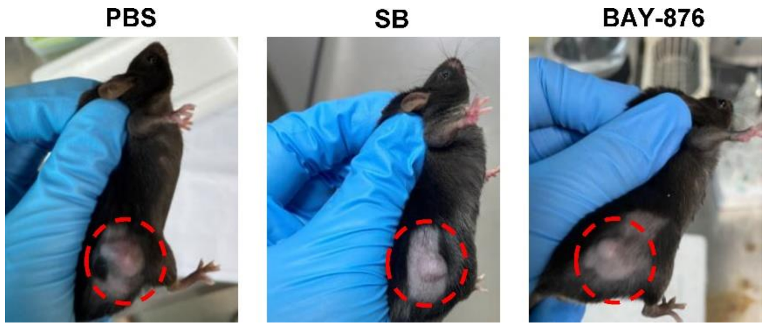


**Fig S4.** Photograph of tumor-bearing mice treated with PBS, SB, and BAY-876 at 21 days.

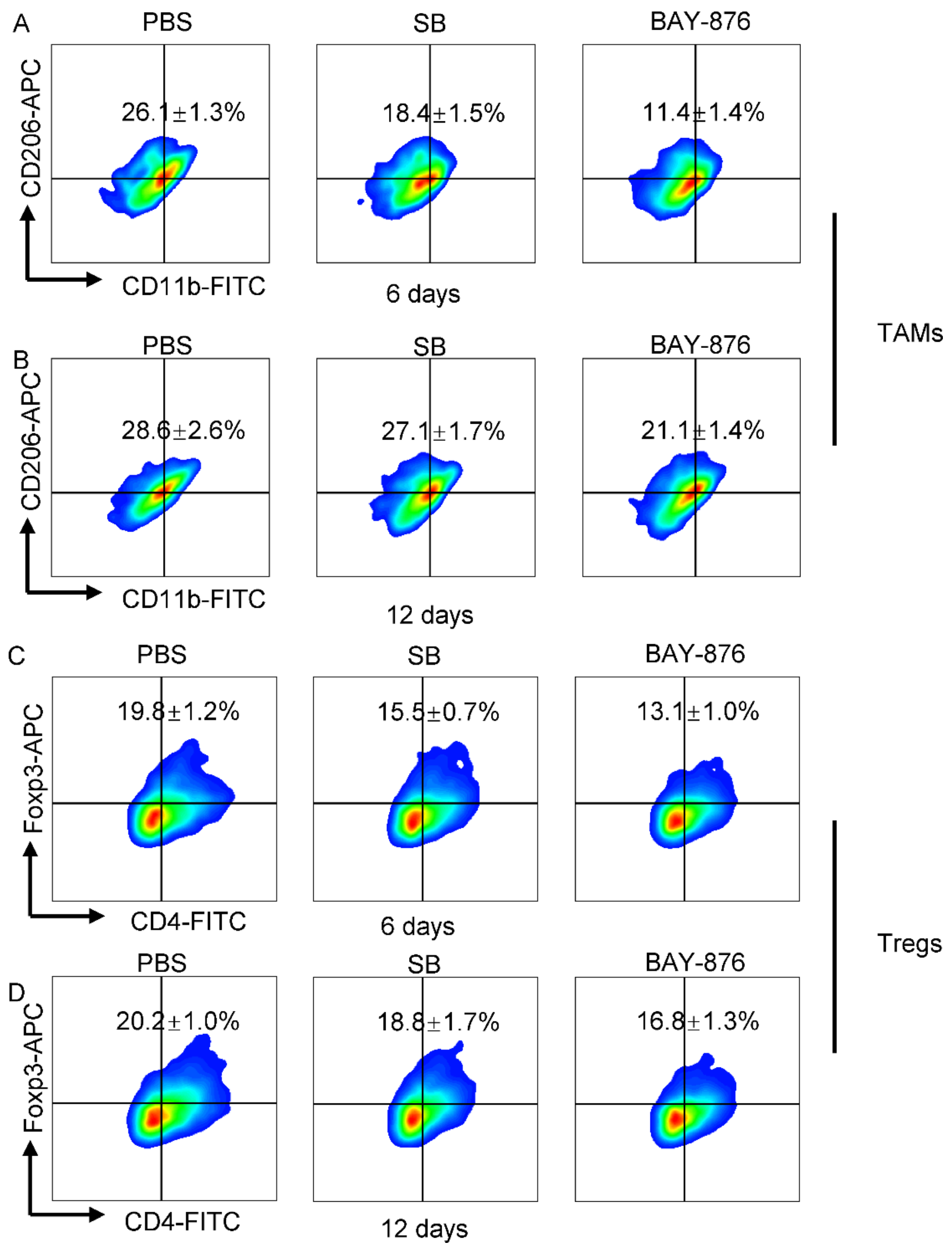

**Fig S5.** FCM examination of the tumor-infiltration of TAMs **(A-B)** ($CD206^+$ gated in $CD45^+CD11b^+$) and Tregs **(C-D)** ($CD4^+Foxp3^+$ gated by $CD3^+$) from mice treated PBS, SB, and BAY-876 at 6 and 12 days.

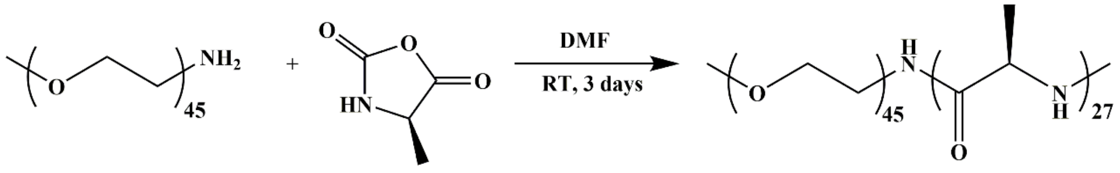


**Fig S6.** Synthesis procedure of PEG-Pla.

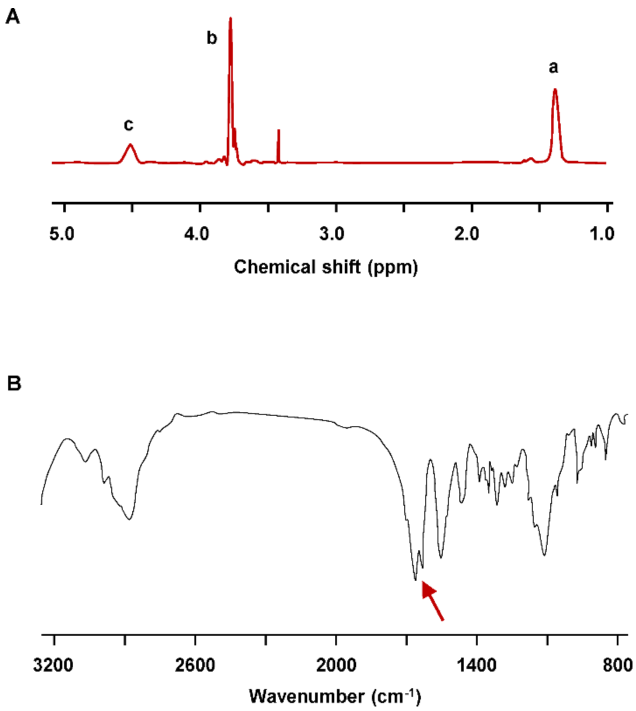


**Fig S7.** $^1H$-NMR spectra and FT-IR spectra of PEG-Pla.

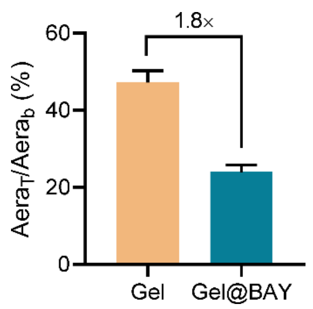


**Fig S8.** Quantified ratio of tumor to brain from mice treated with Gel and Gel@BAY at 21 days.

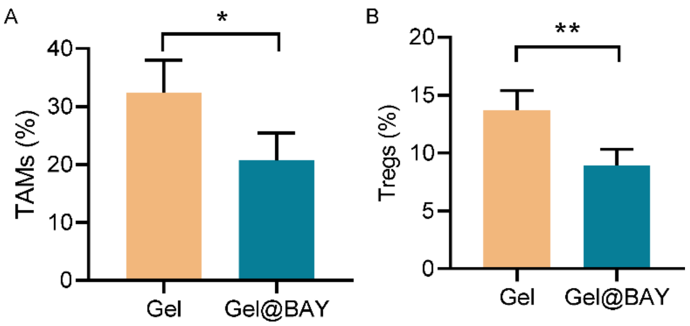


**Fig S9.** Quantification of the tumor-infiltration of TAMs **(A)** and Tregs **(C)** from mice treated Gel and Gel@BAY at 14 days.

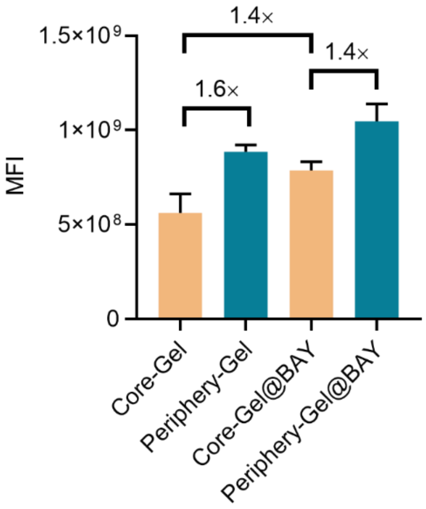


**Fig S10.** Quantitative analysis of the immunofluorescence section (figure 3J) by Image J software.

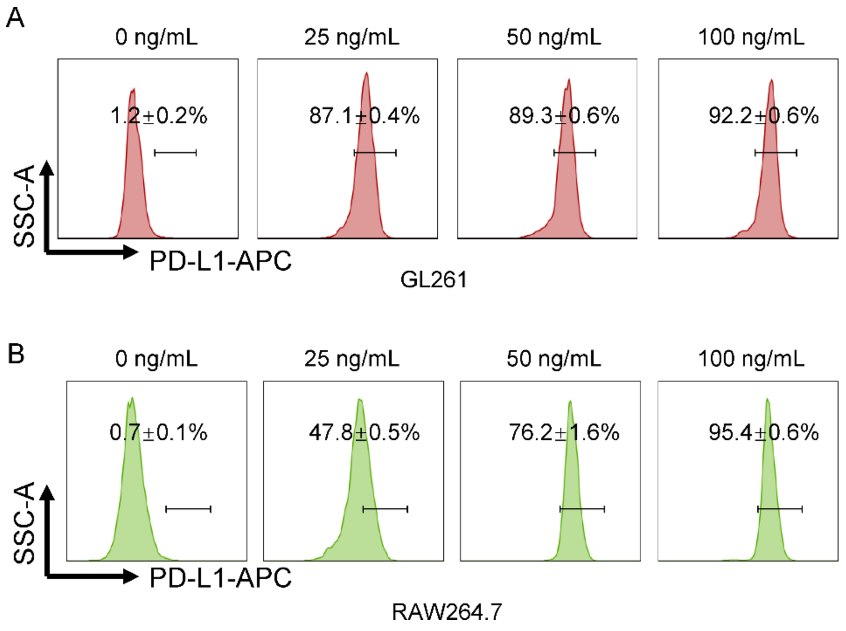


**Fig S11.** FCM examination of the PD-L1 expression of IFN-γ-treated with GL261 and RAW264.7.

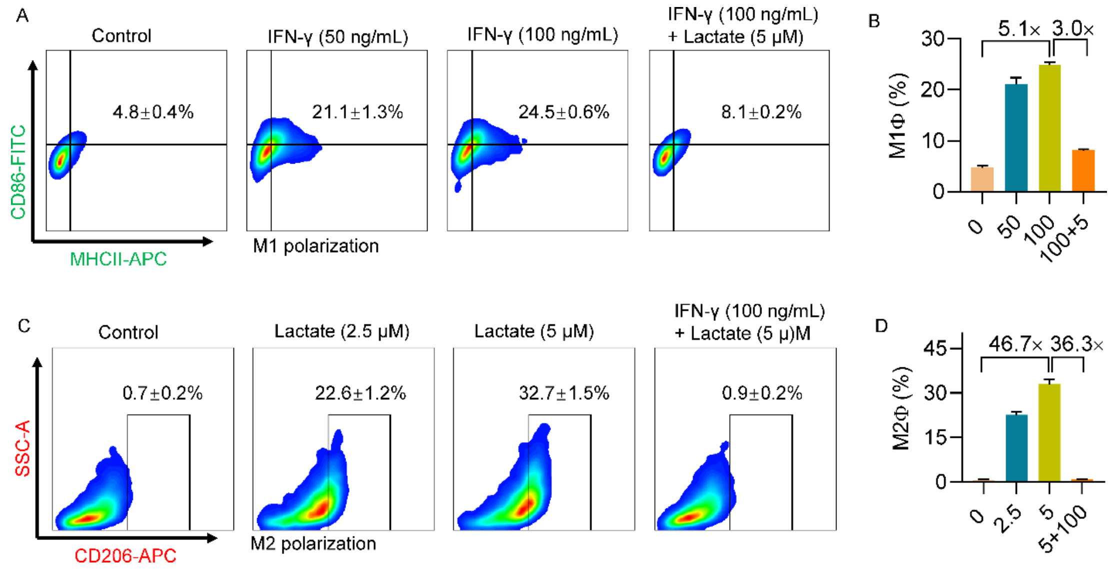


**Fig S12.** FCM analysis and quantification of IFN-γ-induced M1Φ and LA-induced M2Φ in RAW264.7

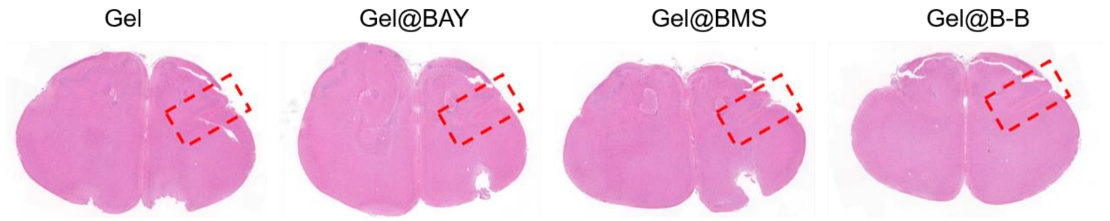


**Fig S13.** H&E analysis of the brain tissues treated by different drug-loaded thermogels for 45 days.

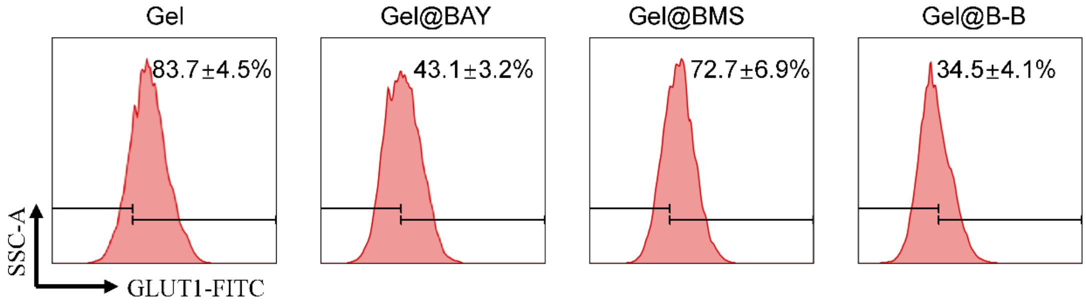


**Fig S14.** FCM examination of $GLUT1^+$ tumor cells ($GLUT1^+$ in $CD45^-$) from mice treated with Gel, Gel@BAY, Gel@BMS, and Gel@B-B.

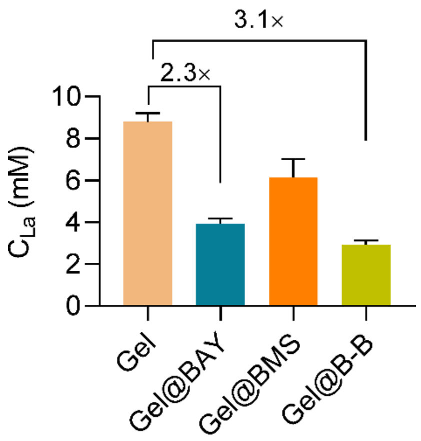


**Fig S15.** Intratumoral LA concentration of mice treated with Gel, Gel@BAY, Gel@BMS, and Gel@B-B.

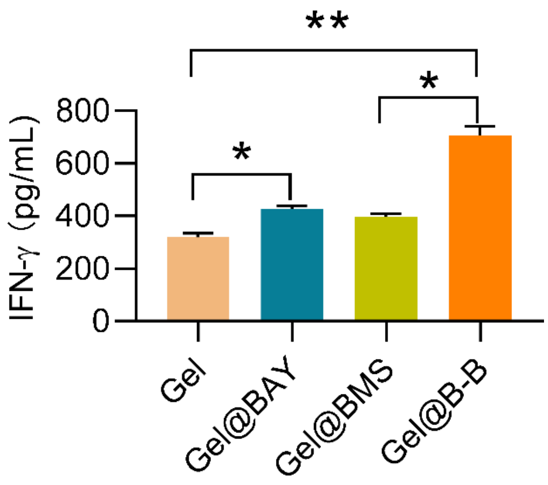


**Fig S16.** Intratumoral IFN-γ concentration of mice treated with Gel, Gel@BAY, Gel@BMS, and Gel@B-B.

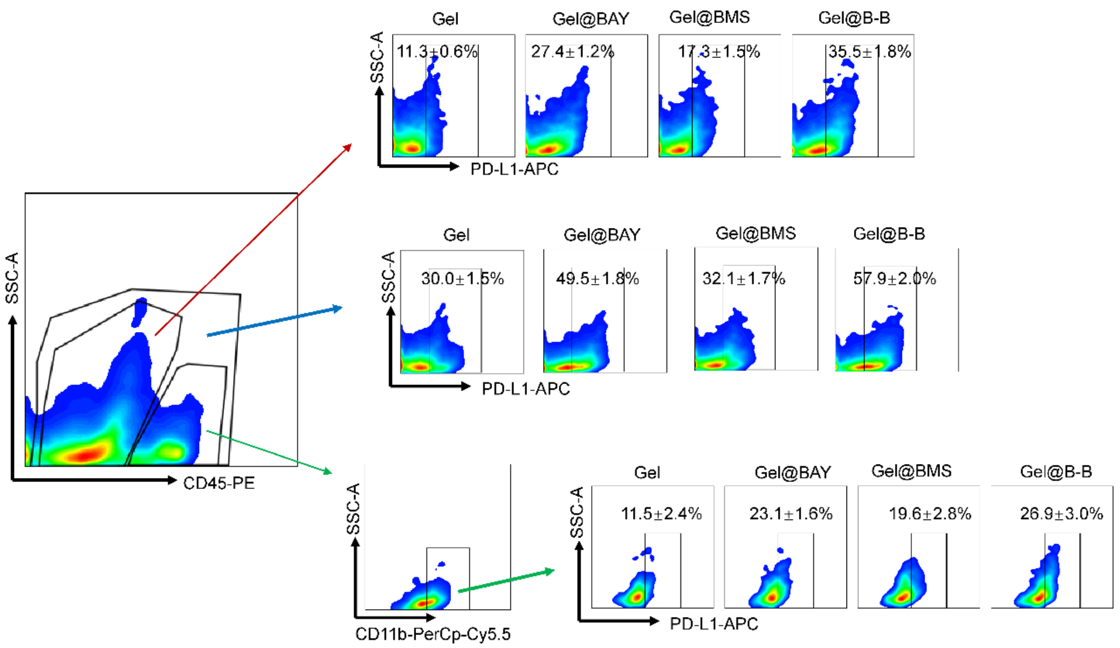


**Fig S17.** FCM examination of PD-L1$^+$ cells (PD-L1$^+$), PD-L1$^+$ tumor cells (PD-L1$^+$ in CD45$^-$), PD-L1$^+$ TAMs (PD-L1$^+$ in CD45$^-$) in tumor tissues from Gel, Gel@BAY, Gel@BMS, and Gel@B-B.

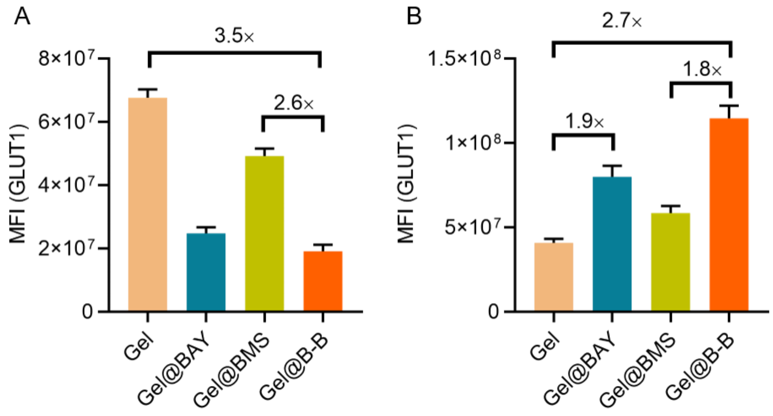


**Fig S18.** Intratumoral IFN-γ concentration of mice treated with Gel, Gel@BAY, Gel@BMS, and Gel@B-B.

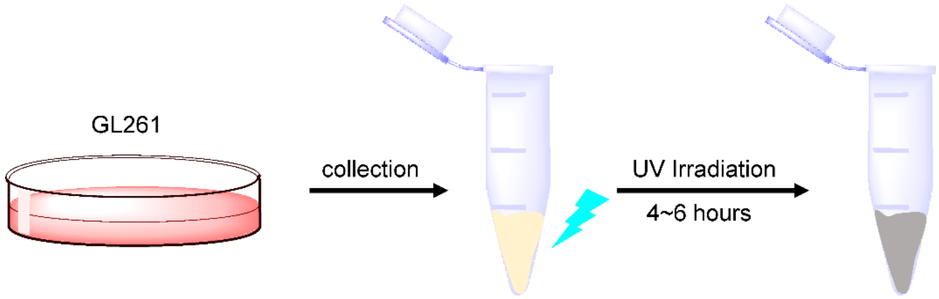


**Fig S19.** Preparation of inactivated GBM vaccines.
